# Inhibitory role of proguanil on the growth of bladder cancer via enhancing EGFR degradation and inhibiting its downstream signaling pathway to induce autophagy

**DOI:** 10.1038/s41419-022-04937-z

**Published:** 2022-05-25

**Authors:** Di Xiao, Xin Hu, Mei Peng, Jun Deng, Sichun Zhou, Simeng Xu, Jingtao Wu, Xiaoping Yang

**Affiliations:** 1 Key Laboratory of Study and Discovery of Small Targeted Molecules of Hunan Province, Department of Pharmacy, School of Medicine, Hunan Normal University, Changsha, Hunan China; 2 Key Laboratory of Protein Chemistry and Developmental Biology of Fish of Ministry of Education, Hunan Normal University, Changsha, Hunan China

**Keywords:** Bladder cancer, Receptor pharmacology

## Abstract

A major reason for the high mortality of patients with bladder cancer (BC) is that chemotherapy and surgery are only effective for very limited patients. Thus, developing novel treatment options becomes an urgent need for improving clinical outcomes and the quality of life for BC patients. Here, we demonstrated that proguanil significantly inhibited the growth of BC in vitro and in vivo. Importantly, our results indicated that the sensitivity of BC cells to proguanil is positively correlated with the expression of epidermal growth factor receptor (EGFR). Mechanistically, proguanil specifically targeted EGFR and promoted EGFR binding to Caveolin-1, enhanced its endocytosis in a Clathrin-independent manner, and then recruited c-Cbl to promote EGFR ubiquitination and degradation through the lysosomal pathway. Further studies suggested that proguanil induced autophagy by destabilizing EGFR and inhibiting its downstream signaling pathway. Thus, this study reveals the novel mechanism of proguanil on anticancer activity and implies the potential benefits of this drug in the treatment of BC.

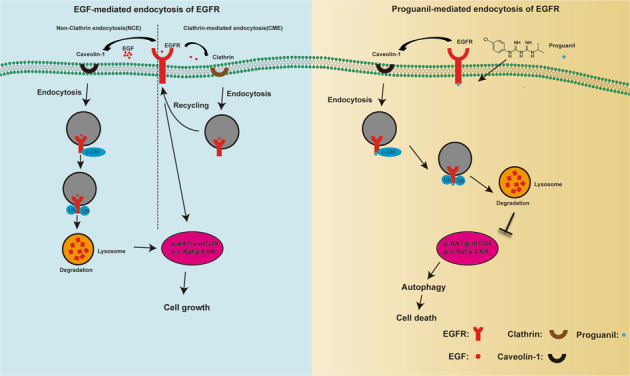

## Introduction

Bladder cancer (BC) is characterized by high invasion, easy metastasis, and high mortality [1]. Surgery and traditional chemotherapy are still the current standard treatment for BC patients [2]. However, the high mortality rate caused by tumor recurrence and metastasis after treatment is a major clinical problem that urgently needs to be resolved [3]. Therefore, exploring new treatments for BC has been a huge clinic demand.

Targeted therapy has achieved great success in the treatment of BC patients. The antibody-drug conjugate enfortumab vedotin improved overall survival and FGFR inhibitor erdafitinib appears active in case of FGFR3 alterations in advanced urothelial carcinoma [4, 5]. Patients who are positive for programmed death ligand 1 and ineligible for using cisplatin may receive immunotherapy (atezolizumab or pembrolizumab) [6]. However, the response rate and overall survival of patients to these targeted drugs are still unsatisfactory. On the other hand, EGFR is one of the major regulatory factors of cell proliferation and metabolism. Chow et al found that EGFR is highly expressed in 72.2% of BC patients [7]. Similarly, Rebouissou et al demonstrated that the EGFR pathway was activated in BC [8]. These results laid the foundation for EGFR as a therapeutic target in BC. However, clinical trials using EGFR-targeting agents either TKI or monoclonal antibodies to treat BC patients have not met the expected treatment effect [9, 10]. Therefore, finding new drugs that target EGFR with mechanism differentiating from TKIs or monoclonal antibody drugs may be benefit for BC patients. In this study, we found for the first time that proguanil (Pro), which has the best bioactivity among biguanide drugs (phenformin, buformin, metformin, and proguanil) in seven BC cell lines [11], specifically targeted EGFR and enhanced its degradation to inhibit the proliferation of BC cells. As we know, when EGFR binds to ligands such as EGF, the downstream signaling pathway of EGFR is activated to facilitate cell growth and differentiation [12, 13], and EGFR itself is internalized through Clathrin-mediated endocytosis (CME) or non-Clathrin endocytosis (NCE) and degraded [14, 15]. Studies have shown that when EGFR is internalized through CME with low concentrations of EGF stimulation, most of EGFR returns to the cell membrane surface by recycling to continue to transmit signals while only a very small part of EGFR is degraded [16]. However, when EGF is in high concentrations, EGFR is internalized through NCE which is regulated by Caveolin-1. Then almost all endocytosed EGFR is ubiquitinated and degraded [16, 17]. The balance between recycling and degradation of EGFR is crucial to ensure the growth of normal cells [18]. EGFR recycling is enhanced or its degradation is weakened with gene mutations in cells, leading to abnormally high expression of EGFR [18, 19], which is the main cause for the pathogenesis of various cancers. Thus, this interesting phenomenon inspires us to deeply explore whether the antitumor effect of proguanil is attributable to the critical role of promoting EGFR endocytosis and degradation. In the current study, we found that proguanil specifically bound to EGFR, enhanced EGFR endocytosis through NCE and degradation in lysosome, then inhibited its downstream signaling pathway, thereby inducing autophagy to block the proliferation of BC cells. Hence, our findings would strongly establish the solid foundation for the potential clinical application of proguanil in BC.

## Results

### Proguanil specifically bound to EGFR and promoted its endocytosis and decay through post-translational regulation

**Fig. 1:**
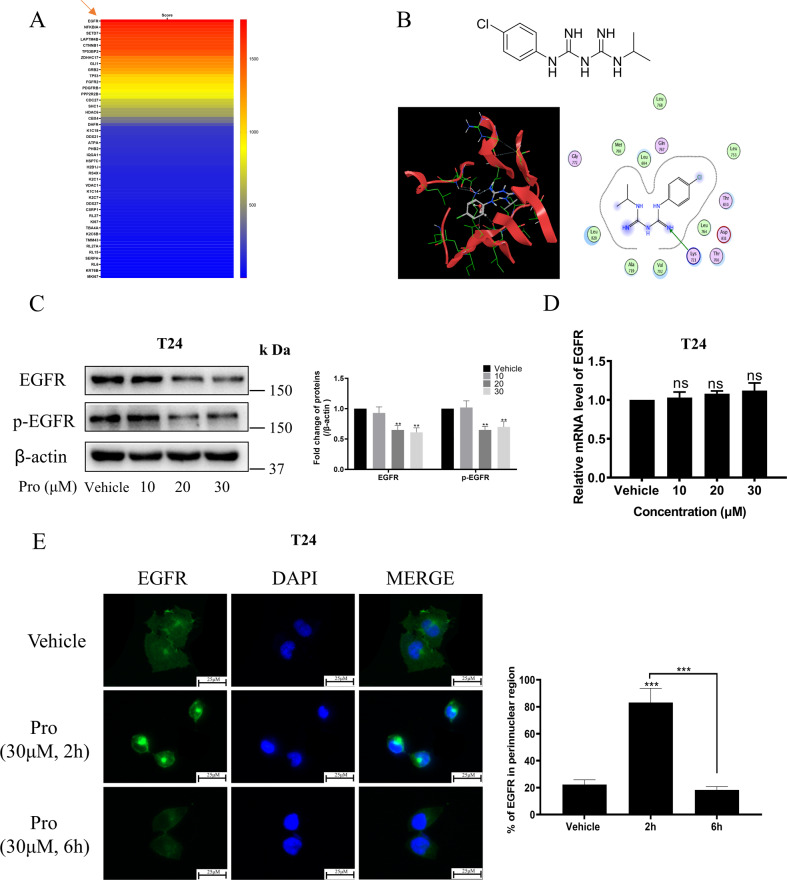
The effect of proguanil on the expression of EGFR. **A** 3D photocrosslinked microarray was used to immobilize proguanil, and then T24 lysates were distributed on the surface of the microarray. The captured target proteins by proguanil on the microarray surface were detected by SPRi and identified by LC-MS. **B** MOE molecular docking (using MOE14.0 software) was used to predict the binding site of proguanil and EGFR (PDB ID − 4HJO). **C** T24 were plated in 6-well plates (3.0 × 10^5^/well). When the cell density reaches 70–80%, cells were starved for 12 h and then treated with proguanil for 6 h, and the expression of p-EGFR and EGFR was detected by WB. **D** T24 were plated in 6-well plates (3.0 × 10^5^ /well). When the cell density reaches 70–80%, cells were starved for 12 h and then treated with proguanil for 6 h, and the expression level of EGFR mRNA was detected by RT-PCR. **E** T24 were plated on glass disks in 12-well plates (2.0 × 10^4^ /well). After 24 h, T24 cells were starved for 12 h and then treated with proguanil, and the changes of EGFR were detected by immunofluorescence. Data are representative of three independent experiments. Error bars represent means ± SD from triplicate experiments. Vehicle control means the concentration of DMSO lower than 0.3% (*P < 0.05, **P < 0.01, ***P < 0.001, ns not significant).

Our previous study found that proguanil significantly inhibited the growth of BC cells [20, 21], while the antitumor mechanism of proguanil has not been reported yet. In this study, in order to understand the antitumor mechanism of proguanil in BC, we first explored the target of proguanil through surface plasmon resonance imaging (SPRi) experiments and found that proguanil strongly binds to EGFR (Fig. 1A). Molecular docking showed that proguanil formed a strong hydrogen bond with the 721 amino acid residue (lysine) of EGFR, and the molecular binding energy was −6.564 kJ /Mol (Fig. 1B). Thus, these results implied that EGFR may be an important target for proguanil, which prompted us to further explore the effect of proguanil on EGFR. Surprisingly, WB results showed that proguanil not only inhibited phosphorylation of EGFR, but also markedly attenuated total EGFR (Fig. 1C). As we all know, blocking EGFR synthesis or promoting EGFR decay are the two main ways to reduce its expression [18, 22]. Thus, we first explored the effect of proguanil on EGFR mRNA. Unexpectedly, the mRNA level of EGFR did not change during proguanil treatment period (Fig. 1D), indicating that proguanil reduced the expression of total EGFR not by inhibiting EGFR mRNA. Next, immunofluorescence results further indicated that there is an even distribution of EGFR throughout the plasma membrane in the absence of proguanil in T24. Interestingly, EGFR was endocytosed and accumulated in a perinuclear location for 2 h treatment of proguanil while decayed after 6 h treatment (Fig. 1E). These results indicated that proguanil enhanced the endocytosis and decay of EGFR in a post-translational manner.

### The expression of EGFR was positively correlated with the poor prognosis of BC patients and the ability of cell proliferation

**Fig. 2:**
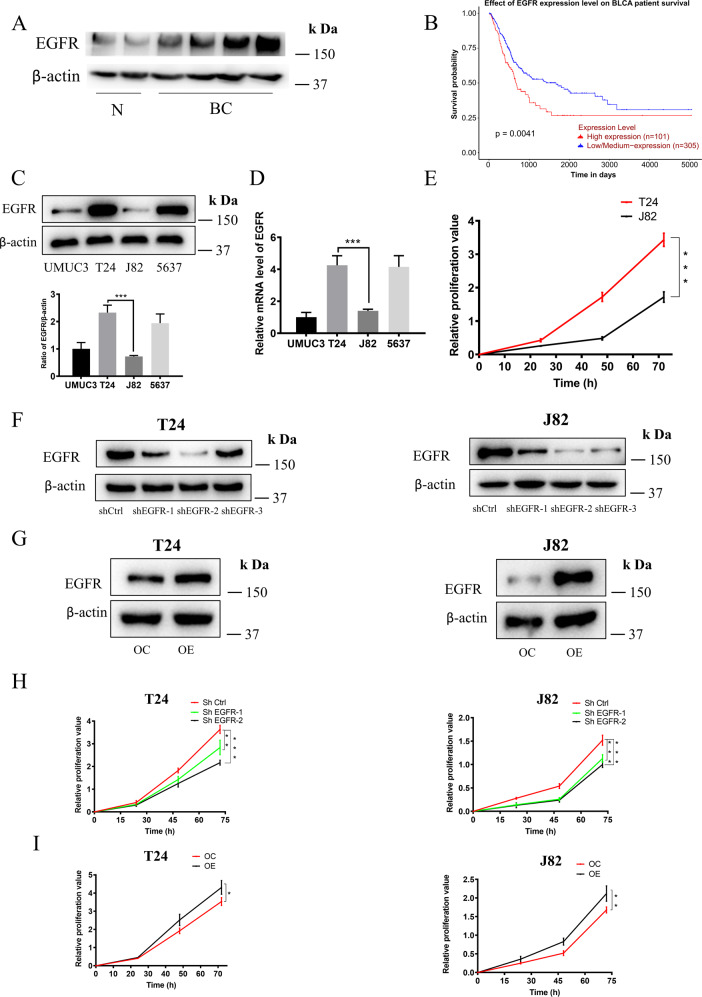
The effect of EGFR expression on patient prognosis and cell proliferation. **A** The expression levels of EGFR in different types of bladder cancer and adjacent tissues were detected by WB. **B** The TCGA database was used to analyze the correlation between EGFR expression and poor prognosis in bladder cancer patients. **C**, **D** Cells were plated in 6-well plates (3.0 × 10^5^ /well) and lysed to collect protein or RNA when density reached 80–90%, the expression of EGFR and mRNA in 4 BC cells were analyzed by WB and RT-PCR. **E** Cells were plated in 96-well plates (6.0 × 10^3^ /well), The proliferation of T24 and J82 cells was detected at specified times by MTT assay. EGFR stable knockdown (**F**) and overexpressed (**G**) cell lines were constructed by lentiviral vector plasmid as described in “Materials and methods”, then cells were plated in 6-well plates (3.0 × 10^5^ /well) and lysed to collect protein when density reached 80–90%. The expression of EGFR was detected by WB. **H**, **I** Cells were plated in 96-well plates (6.0 × 10^3^/well), the proliferation of cell lines were detected at specified times by MTT assay. Data are representative of three independent experiments. Error bars represent means ± SD from triplicate experiments. Vehicle control means the concentration of DMSO lower than 0.3% (*P < 0.05, **P < 0.01, ***P < 0.001).

To explore the role of EGFR in the progression of BC, we examined the expression of EGFR in BC patient specimens. As show in Fig. 2A, EGFR was significantly higher in BC tissues compared with cancer free adjacent tissues. These results are consistent with previous Mooso et al’s observations [23]. After confirming the high expression of EGFR in BC, we further analyzed the influence of EGFR expression on the survival time of patients by TCGA database. These facts imply a positive correlation between EGFR expression and poor prognosis in patients (Fig. 2B). Subsequently, we analyzed the basic level of EGFR protein and mRNA in four BC cell lines by WB and RT-PCR. As shown in Fig. 2C, D, we found that the expression of EGFR protein and mRNA are highest in T24 and lowest in J82. MTT assay demonstrated that T24 (EGFR high expression) proliferated faster than J82 (EGFR low expression) (Fig. 2E). To further explore the relationship between EGFR expression and the proliferation rate of cancer cell lines, cell lines with stable knockdown (sh) or overexpression EGFR (OE) were constructed by lentiviral transfection and verified by WB (Fig. 2F, G). MTT results showed that shEGFR-1, shEGFR-2 with better silencing effect significantly reduced cell growth (Fig. 2H) and OE promoted cell growth (Fig. 2I). these results indicated that the high expression of EGFR was closely related to poor prognosis of patients and the proliferation of BC cells.

### The expression of EGFR is positively correlated with the sensitivity of cancer cells to proguanil

**Fig. 3:**
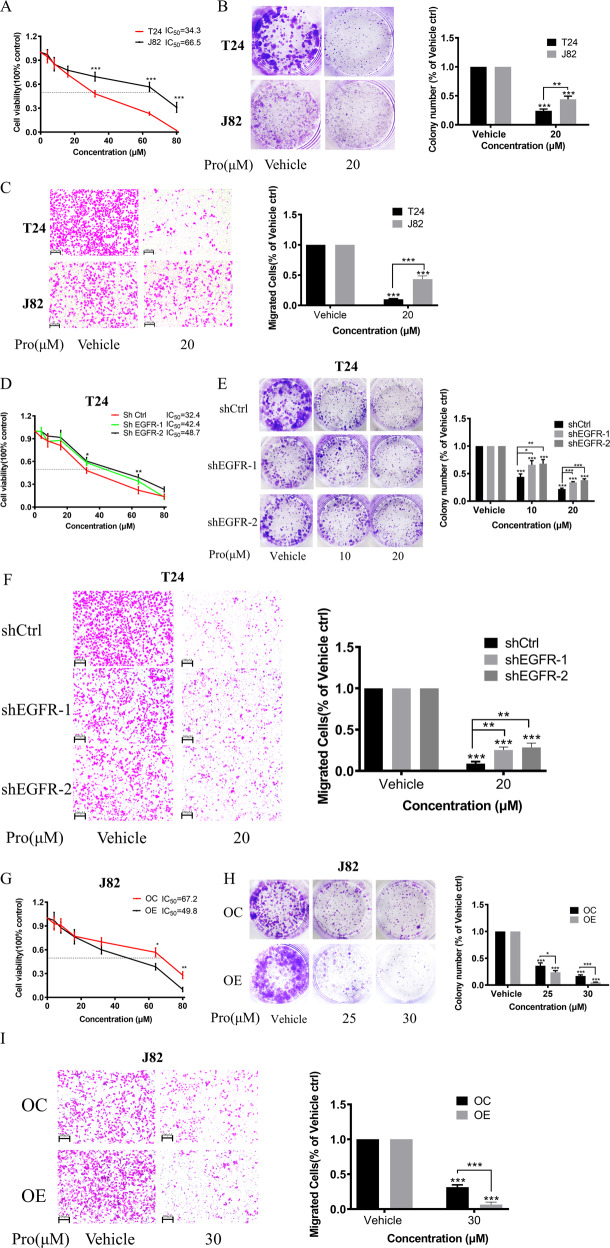
The relationship of EGFR expression and the sensitivity of BC cells to proguanil. **A**–**C** The inhibitory effects of proguanil on proliferation and migration of T24 and J82 cell lines were compared by MTT, colony formation and transwell assay. **D**–**F** The inhibitory effects of proguanil on proliferation and migration of shCtrl, shEGFR-1 and shEGFR-2 cell lines were compared by MTT, colony formation and transwell assay. **G**–**I** The inhibitory effects of proguanil on proliferation and migration of OC-J82 and OE-J82 were compared by MTT, colony formation and transwell assay. See “Materials and methods” for more experimental details. Data are representative of three independent experiments. Error bars represent means ± SD from triplicate experiments. Vehicle control means the concentration of DMSO lower than 0.3% (*P < 0.05, **P < 0.01, ***P < 0.001).

To further confirm whether proguanil inhibits the progress of BC cells by targeting EGFR, we first compared the inhibitory activity of proguanil on T24 (EGFR high expression) and J82 (EGFR low expression) by MTT, colony formation, and transwell assays. Our results showed that proguanil has a more significant inhibitory effect on T24 than that on J82 (Fig. 3A–C). Next, we investigated the effect of proguanil on the proliferation of cells by regulating EGFR levels. Interestingly, after stably knocking down the expression of EGFR in T24 cells, the IC_50_ of proguanil in shEGFR-1-T24 (IC_50_ = 42.7 μM) and shEGFR-2-T24 (IC_50_ = 48.7 μM) increased by 10.3 μM and 16.3 μM respectively compared with shCtrl-T24 (IC_50_ = 32.4 μM) (Fig. 3D). Similarly, knocking down EGFR significantly reduced the inhibitory effect of proguanil on colony formation and migration, which were detected by the colony formation and transwell assays (Fig. 3E, F). In contrast, IC_50_ in overexpressed EGFR J82 cells (OE-J82) was reduced to 49.8 μM compared to 67.2 μM in overexpressed control-counterparts (OC-J82) (Fig. 3G). Further, colony formation and transwell experiments demonstrated that the inhibitory effect of proguanil on OE-J82 cells became stronger than OC-J82 cells (Fig. 3H, I). Taken together, these results indicated that the expression of EGFR in BC cells has a profoundly positive correlation with its sensitivity to proguanil.

### Proguanil-enhanced EGFR endocytosis and decay in Clathrin-independent manner

**Fig. 4:**
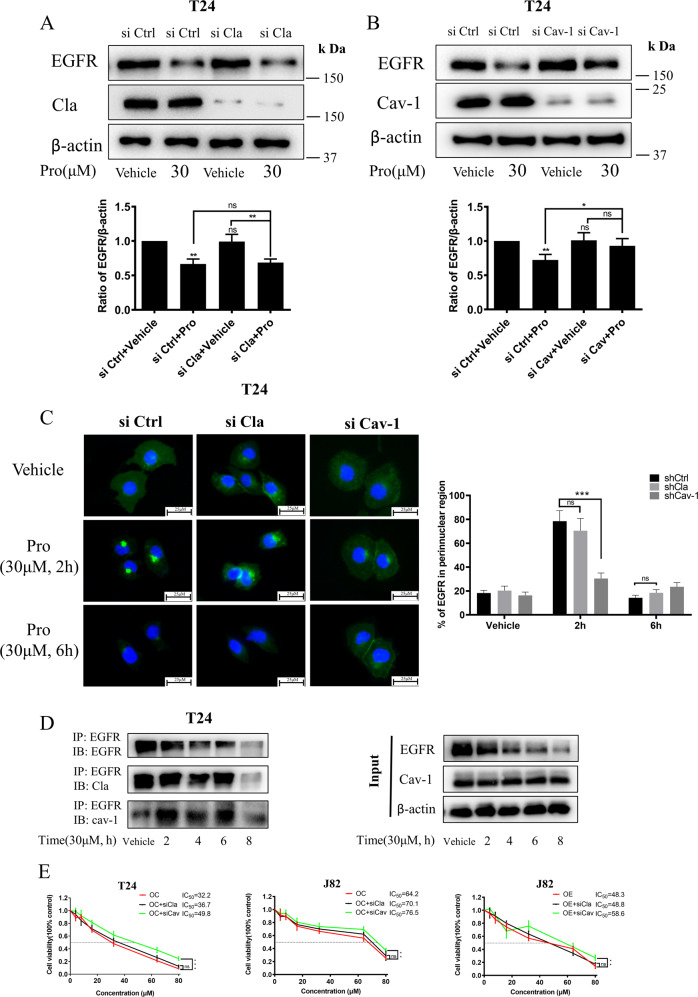
Study on the mechanism of proguanil-enhanced EGFR endocytosis. **A**, **B** T24 were plated in 6-well plates (2.0 × 10^5^/well) and transfected with corresponding siRNA as described in “Materials and methods”. When the cell density reaches 70–80%, cells were starved for 12 h and then treated with proguanil for 6 h. The effect of proguanil on the expression of EGFR was detected by WB. **C** After si Clathrin or si Caveolin-1 as described in “Materials and methods”, T24 were plated on glass disks in 12-well plates (2.0 × 10^4^/well), starved for 12 h and then treated with proguanil, and the changes of EGFR were detected by immunofluorescence. **D** When the density of T24 reaches 70–80% in 75 cm^2^ vented culture flasks, cells starved for 12 h and then treated with proguanil. Then protein lysates were immunoprecipitated with EGFR antibody. The immunoprecipitate were tested by WB with antibodies to EGFR, Clathrin and Caveolin-1. **E** Cells were transfected with corresponding siRNA as described in “Materials and methods” and then plated in 96-well plates (6.0 × 10^3^/well), the bioactivity of proguanil after si Clathrin or si Caveolin-1 was detected by MTT assay. Data are representative of three independent experiments. Error bars represent means ± SD from triplicate experiments. Vehicle control means the concentration of DMSO lower than 0.3% (*P < 0.05, **P < 0.01, ***P < 0.001, ns not significant).

We have demonstrated that proguanil promoted the endocytosis and decay of EGFR. However, the specific mechanism remains elusive. Since CME and NCE are two manners of EGFR endocytosis [14, 15], we silenced either CME-associated protein Clathrin (Cla) or NCE-associated protein Caveolin-1 (Cav-1) to explore whether the alteration of these two proteins affects the effect of proguanil on EGFR. Interestingly, EGFR decay promoted by proguanil is not affected by silencing Clathrin while the ability of proguanil to promote EGFR decay was significantly reduced by silencing Caveolin-1 (Fig. 4A, B). It should be noted that either silencing Clathrin or Caveolin-1 has no effect on the expression of EGFR. Meanwhile, immunofluorescence results further demonstrated that EGFR endocytosis accumulated in a perinuclear location and then decay promoted by proguanil were not affected by silencing Clathrin (Fig. 4C). However, after silencing Caveolin-1, most of the EGFR is still evenly distributed throughout the plasma membrane without endocytosis and decay after proguanil treatment (Fig. 4C). More profoundly, IP results showed that the binding of EGFR to Caveolin-1 but not to Clathrin was significantly enhanced after proguanil treatment for 2 h (Fig. 4D). Consistently, MTT results showed that the IC_50_ of proguanil in T24, J82 and OE-J82 cells were not significantly modified compared with IC_50_ in silencing Clathrin cells, while significantly higher concentrations of proguanil were needed to achieve the IC_50_ in silencing Caveolin-1 cells (Fig. 4E). Taken together, these results stated above suggest that proguanil-enhanced EGFR endocytosis in a Clathrin-independent manner.

### Proguanil recruited c-Cbl to promote EGFR ubiquitination and degradation in the lysosome

**Fig. 5:**
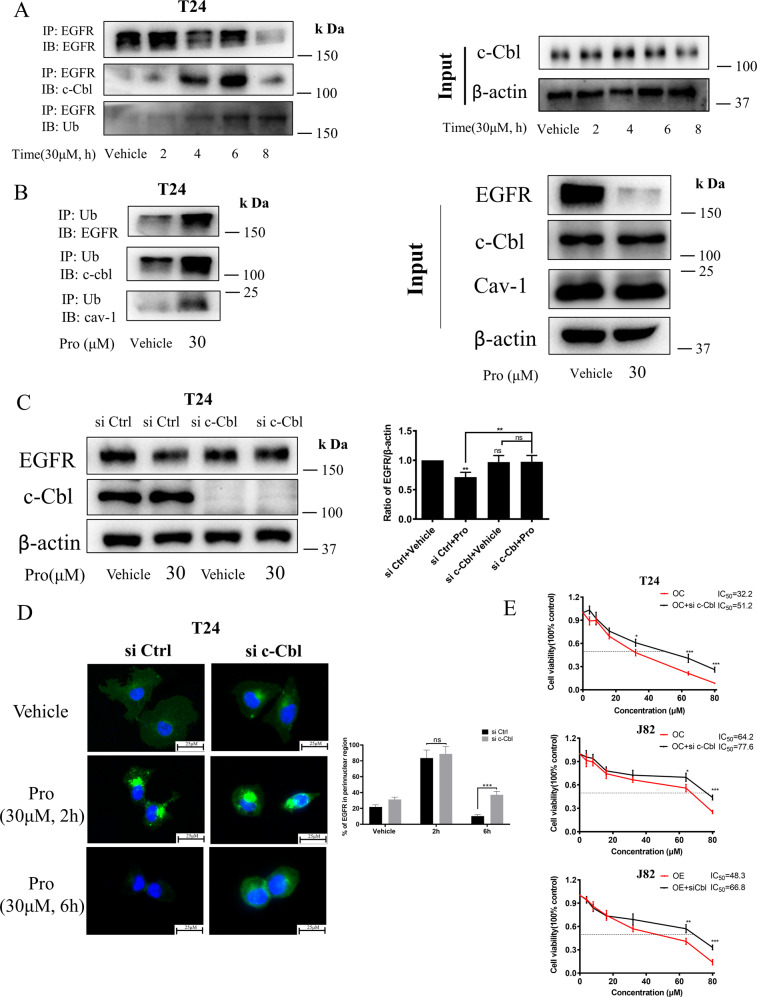
Study on the mechanism of proguanil-enhanced EGFR ubiquitination and degradation. **A** When the density of T24 reaches 70–80% in 75 cm^2^ vented culture flasks, cells starved for 12 h and then treated with proguanil. Then protein lysates were immunoprecipitated with EGFR antibody. The immunoprecipitates were tested by WB with antibodies to EGFR, c-Cbl and Ub. **B** When the density of T24 reaches 70–80% in 75 cm^2^ vented culture flasks, cells starved for 12 h and then treated with proguanil. Then protein lysates were immunoprecipitated with Ub antibody. The immunoprecipitates were tested by WB with antibodies to EGFR, c-Cbl and Cav-1. **C** T24 were plated in 6-well plates (2.0 × 10^5^ /well) and transfected with corresponding siRNA as described in “Materials and methods”. When the cell density reaches 70–80%, cells were starved for 12 h and then treated with proguanil for 6 h. The effect of proguanil on the expression of EGFR and c-Cbl were detected by WB. **D** After si c-Cbl as described in “Materials and methods”, T24 were plated on glass disks in 12-well plates (2.0 × 10^4^/well), starved for 12 h and then treated with proguanil for 6 h. The effects of proguanil on EGFR endocytosis and expression were detected by immunofluorescence. **E** Cells were transfected with corresponding siRNA as described in “Materials and methods” and then plated in 96-well plates (6.0 × 10^3^ /well). The bioactivity of proguanil on cells after si c-Cbl was detected by MTT assay. Data are representative of three independent experiments. Error bars represent means ± SD from triplicate experiments. Vehicle control means the concentration of DMSO lower than 0.3% (*P < 0.05, **P < 0.01, ***P < 0.001, ns not significant).

The next question is, after proguanil-enhanced the endocytosis of EGFR, how does it promote EGFR decay? Studies have shown that EGFR ubiquitin degradation is a key step in its decay [24]. Therefore, we explored whether proguanil could further promote EGFR ubiquitin degradation after enhancing EGFR endocytosis. IP results showed that the binding of both Ub and c-Cbl to EGFR gradually increased after proguanil treatment for 4 h (Fig. 5A, B). c-Cbl is an E3 ubiquitin ligase that promotes substrate to be labeled by ubiquitin to promote the protein degradation [25]. Importantly, the ability of proguanil to promote EGFR decay was significantly reduced by silencing c-Cbl while silencing c-Cbl itself has no effect on the expression of EGFR (Fig. 5C). After silencing c-Cbl, immunofluorescence results indicated that EGFR was in an endocytosed and perinuclear aggregated status for 2 h proguanil treatment but in reduced degradation for 6 h proguanil treatment compared with si Ctrl cells (Fig. 5D), indicating that c-Cbl is indispensable in the process of proguanil-enhanced EGFR ubiquitin degradation. Consistently, MTT results showed that silencing c-Cbl significantly reduced the inhibition ability of proguanil on T24, J82, and OE-J82 (Fig. 5E). Interestingly, we found that Caveolin-1 binds to EGFR earlier, while Ub binds to EGFR later after proguanil treatment (Figs. 4D and 5A), indicating that proguanil-enhanced EGFR endocytosis first, and then promotes EGFR ubiquitin degradation. We next explored whether proguanil affected the downstream signaling pathways of EGFR after promoting it degradation. WB analysis indicated that the phosphorylation of AKT/mTOR and c-Raf/ ERK was significantly decreased after proguanil treatment (SFig. 1A). Moreover, Chloroquine, a lysosomal inhibitor, was used to destroy the lysosome to probe the location of EGFR degradation. The results demonstrated that the abilities that proguanil down-regulated EGFR and p-AKT/p-mTOR/p-c-Raf/p-ERK were significantly reduced in the presence of Chloroquine, implying that proguanil-enhanced EGFR degradation is located in lysosome (SFig. 1B). We then measured the endocytosis of EGFR and the expression of its downstream signaling pathway after EGF stimulation. The results showed that EGF promoted EGFR endocytosis and activates the phosphorylation of AKT/mTOR and c-Raf/ERK1/2 (SFig. 2A, B), while proguanil significantly inhibited EGF-induced endocytosis and accelerated the degradation of EGFR to reverse or attenuate EGF-induced activation of p-AKT/ p-mTOR and p-c-Raf/ p-ERK1/2 (SFig. 2C, D). These results indicated that proguanil-enhanced EGFR degradation in lysosome leads to the decrease of membrane EGFR, causing the loss of EGF binding to EGFR.

### Proguanil induced autophagy in BC cells

**Fig. 6:**
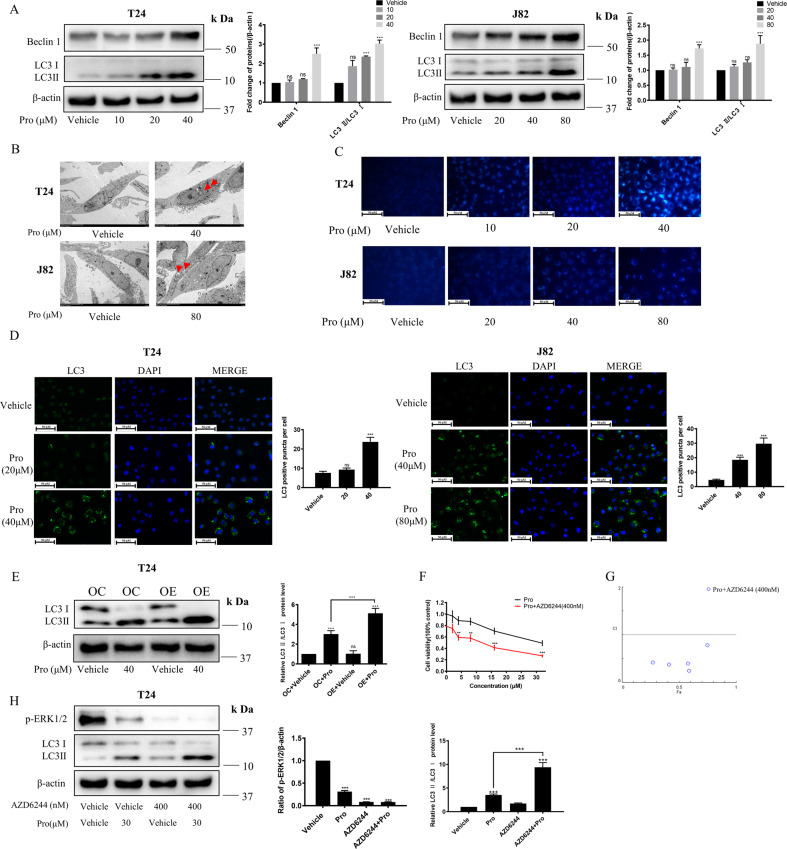
Proguanil induces autophagy in BC cells. **A** T24, J82 were plated in 6-well plates (3.0 × 10^5^/well). When the cell density reaches 70–80%, cells were treated with proguanil for 12 h, and the protein expressions of LC3 and Beclin-1 were detected by WB. **B** When the density of T24 and J82 reaches 70–80% in 75 cm^2^ vented culture flasks, cells were treated with proguanil for 12 h. Subsequently, cells are then fixed with electron microscope fixative and observed under a transmission electron microscope (Red arrows indicate autophagosomes). **C** T24, J82 were plated on glass disks in 12-well plates (8.0 × 10^4^ /well) and treated with proguanil for 12 h. Subsequently, cells washed with wash buffer and stained with MDC as described in “Materials and methods”, and then observed under an inverted fluorescence microscope. **D** T24, J82 were plated on glass disks in 12-well plates (2.0 × 10^4^/well), After 24 h, cells were treated with proguanil for 12 h. The effect of proguanil on LC3 was detected by immunofluorescence. **E** T24, OE-T24 were plated in 6-well plates (3.0 × 10^5^ /well). When the cell density reaches 70–80%, cells were treated with proguanil for 12 h, the effect of proguanil on the expression of LC3 was detected by WB. **F** T24 were plated in 96-well plates (6.0 × 10^3^/well) and pretreated with 400 nm AZD6244 for 12 h before exposure to increasing concentrations of proguanil for an additional 72 h, the combined effect of proguanil and AZD6244 was detected by MTT assay. **G** Combination index (CI) among the combinations of two drugs was calculated using CompuSyn software. if CI > 1, it denotes antagonism; if CI < 1, it denotes synergism. CI values in all of combinations were less than 1, indicating synergism. **H** T24 were plated in 6-well plates (3.0 × 10^5^/well). When the cell density reaches 70–80%, cells were pretreated with 400 nm AZD6244 for 12 h before exposure to proguanil for an additional 12 h, the protein expressions of p-ERK1/2 and LC3 were detected by WB. Data are representative of three independent experiments. Error bars represent means ± SD from triplicate experiments. Vehicle control means the concentration of DMSO lower than 0.3% (*P < 0.05, **P < 0.01, ***P < 0.001, ns not significant).

Previous studies have shown that EGFR endocytosis is involved in autophagy regulation [26, 27]. Emerging evidence also suggest that EGFR and its downstream signaling pathways are closely related to autophagy regulation in various cancers, and targeting EGFR-mediated autophagy is a potential strategy for cancer treatment [28]. Thus, we further explored whether proguanil regulates the level of autophagy. WB results indicated that the expression of autophagy marker proteins Beclin-1 and LC3-II dramatically increased after proguanil treatment (Fig. 6A). Meanwhile, transmission electron microscopy further detected autophagosomes in proguanil-treated cells, while the untreated BC cells showed normal cytoplasm, nucleus, organelles without production of autophagic vesicles (Fig. 6B). Furthermore, MDC and immunofluorescence assays revealed that proguanil induced autophagy in T24 and J82 cells (Fig. 6C, D). Interestingly, the ability of proguanil to induce autophagy in T24 (EGFR high expression) is significantly stronger than that in J82 (EGFR low expression) (Fig. 6A–D), and overexpression of EGFR in T24 significantly enhanced proguanil-induced autophagy (Fig. 6E). To further explore the relationship between proguanil-induced autophagy and EGFR downstream signaling pathways, MK-2206, AY-22989, AZD6244 were used to inhibited the phosphorylation of AKT, mTOR, ERK1/2 before proguanil treatment. As expected, the inhibition of AKT, mTOR, ERK1/2 all enhanced the inhibitory effects of proguanil on cell viability (Fig. 6F, G, SFig. 3A–D). Moreover, compared to cells treated with proguanil alone, the expression of LC3-II was further increased after proguanil combined with MK-2206, AY-22989, AZD6244, respectively (Fig. 6H, SFig. 3E, F). All these data suggested that proguanil induced autophagy by inhibiting EGFR and its downstream signaling pathways.

### Inhibition of autophagy by 3-MA decreased the anti-proliferation activity of proguanil in BC cells

**Fig. 7:**
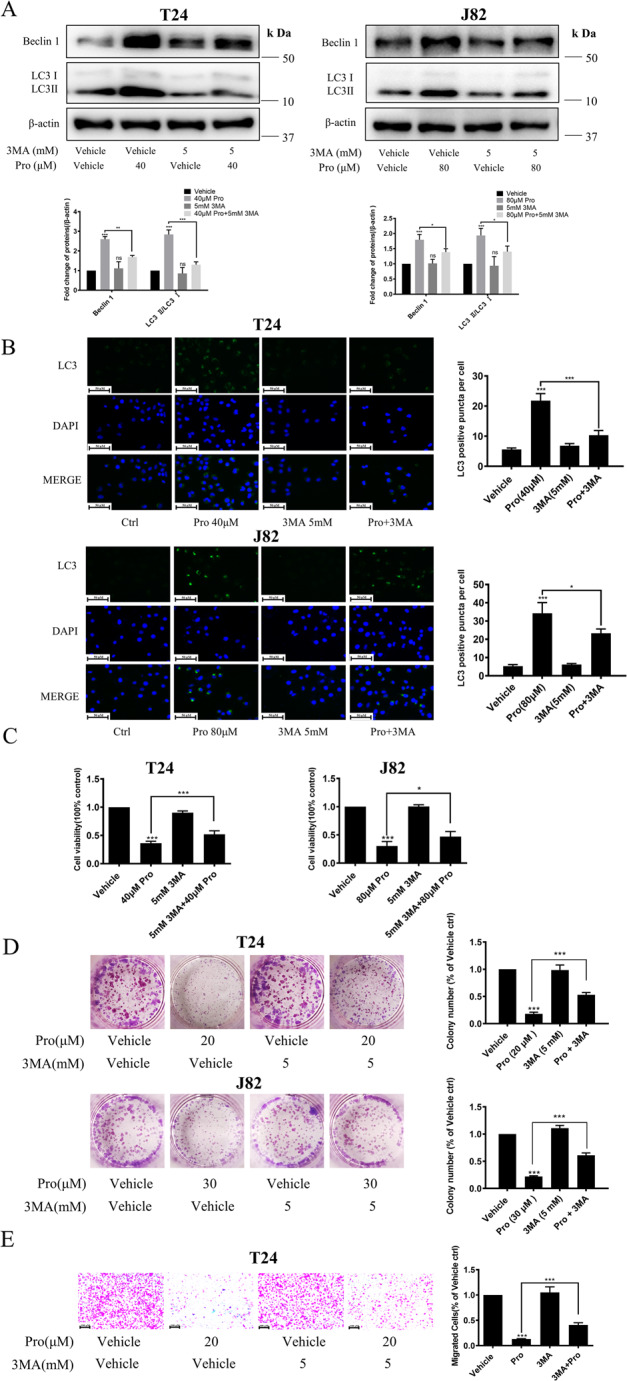
Inhibition of autophagy by 3-MA reduced the activity of proguanil in BC cells. **A** T24, J82 were plated in 6-well plates (3.0 × 10^5^ /well). When the cell density reaches 70–80%, cells were treated with 3-MA (5 mM) and proguanil for 12 h. Western blotting was used to detect the protein expression of LC3 and Beclin-1. **B** T24, J82 were plated on glass disks in 12-well plates (2.0 × 10^4^/well), After 24 h, cells were treated with 3-MA (5 mM) and proguanil, immunofluorescence was used to detect the protein expression of LC3. **C** Cells were plated in 96-well plates (6.0 × 10^3^/well), After 12 h, cells were treated with 3-MA (5 mM) and proguanil, the inhibitory effects of proguanil and 3-MA on proliferation were detected by MTT. **D** Cells were plated in 24-well plates (2.0 × 10^3^/well), After 12 h, cells were treated with 3-MA (5 mM) and proguanil, the he inhibitory effects of proguanil and 3-MA on proliferation were detected by colony formation assay. **E** The inhibitory effects of proguanil and 3-MA on migration were detected by transwell as described in “Materials and methods”. Data are representative of three independent experiments. Error bars represent means ± SD from triplicate experiments. Vehicle control means the concentration of DMSO lower than 0.3% (*P < 0.05, **P < 0.01, ***P < 0.001, ns not significant).

In order to further confirm proguanil inhibited the proliferation of BC by inducing autophagy, we used a specific autophagy inhibitor 3-methyladenine (3-MA), which can inhibit the formation of autophagosomes in the initial stage of autophagy, to explore whether treatment of 3-MA has any alleviating effect on antitumor activity and autophagy-related protein regulation of proguanil. As shown in Fig. 7A, after 3-MA treatment, the upregulation of Beclin-1 and LC3-II induced by proguanil was significantly reduced. Meanwhile, the immunofluorescence images of the LC3 consistently demonstrated the similar trends (Fig. 7B). In addition, MTT results showed that after 3-MA blocked autophagy, the ability of proguanil to inhibit the proliferation of BC cells was reduced (Fig. 7C). Consistently, the inhibitory effect of proguanil on the proliferation and migration of BC cells was also reduced after autophagy was suppressed by 3-MA (Fig. 7D, E). These results above indicated that proguanil inhibited proliferation and migration of BC cells via autophagy-associated events.

### In vivo effect of proguanil on the growth of xenograft tumor

**Fig. 8:**
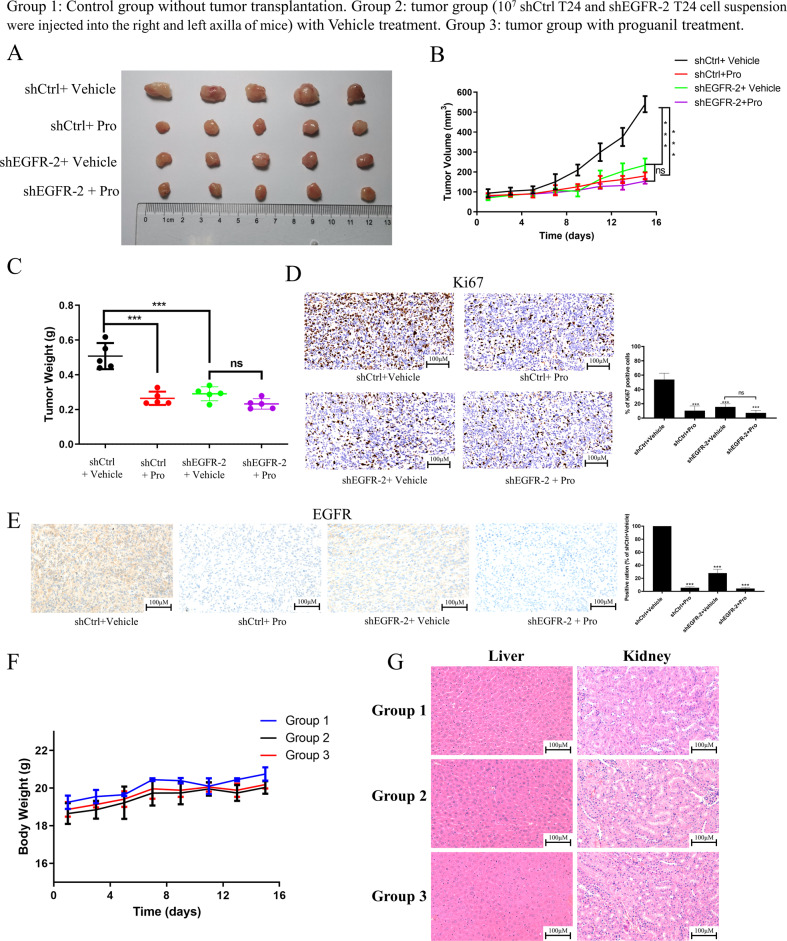
Proguanil-enhanced EGFR degradation to inhibit the growth of xenograft tumor in vivo. **A** 10^7^ T24 and shEGFR-2 T24 cell suspension were injected into the right and left axilla of mice in group 2 and group 3. When the tumor volume reached 70–100 mm^3^, mice in group 3 were treated with proguanil. After treated 14 days, tumors were removed and photographed. **B** The tumor volume of each mouse was measured every two days, and the mean volume of each group tumor was calculated to create the figure. **C** Statistical analysis of tumor weight. **D** Ki-67 was used to analyze the proliferation of xenograft tumor. **E** The expression of EGFR in tumor tissues was detected by immunohistochemistry. **F** Changes of each group mice weight. **G** HE staining of liver and kidney organs of each group mice. All in vitro experiments are representative of three independent experiments. Error bars represent means ± SD. Vehicle control(2% PEG-400 + 2% Tween-80 + 96% PBS). (*P < 0.05, **P < 0.01, ***P < 0.001, ns not significant).

Since proguanil has excellent inhibitory effect on BC cell growth, we continue to evaluate the antitumor activity of proguanil in vivo. When the tumor volume reached 70–100 mm^3^, mice were treated with proguanil. As shown in Fig. 8A, B and SFig. 4A, B, the volume of T24 xenograft tumors was distinctly decreased after proguanil treatment. More interestingly, no difference of tumor volume was detected between shEGFR-2-T24 untreated group and shEGFR-2-T24 proguanil-treated groups, indicating the critical role of EGFR in inhibitory effect of proguanil (Fig. 8A, B). Consistently, compared with untreated group, the weight of T24 tumors in proguanil-treated group was reduced by 47% (Fig. 8C, SFig. 4C), while no significant difference between in the weight of shEGFR-2-T24 untreated tumors and shEGFR-2-T24 proguanil-treated tumors (Fig. 8C). Therefore, these results strongly suggest that proguanil may inhibit the growth of tumor xenografts by targeting EGFR. Immunohistochemical results of Ki-67 showed that the number of Ki-67 positive tumor cells in the proguanil treatment group was significantly reduced compared with untreated group (Fig. 8D, SFig. 4D). In addition, EGFR immunohistochemistry results further confirmed that proguanil dramatically downregulates the expression of EGFR in vivo (Fig. 8E). Together, all data strongly suggested that proguanil suppressed xenograft tumors growth by targeting EGFR.

On the other hand, the role of autophagy on antitumor effect of proguanil was explored in vivo by using the xenograft mice as well. As shown in SFig. 4A–C, after the treatment of 3-MA, the specific autophagy inhibitor, the effect of proguanil on T24 xenograft tumor was significantly reduced. Moreover, the number of Ki-67 positive cells in tumor tissues was increased in the presence of autophagy inhibitor, 3-MA in proguanil-treated group compared with proguanil treatment alone (SFig. 4D). In addition, LC3 immunohistochemistry further revealed that proguanil could up-regulate the expression of this autophagy biomarker protein (SFig. 4E). Taken together, these results indicated that proguanil inhibited tumor progression by inducing autophagy.

Since proguanil was marketed as the first-line drug for treating malarial with the advantages of low toxicity, we paid attention to body weight change of mice used in this study. We noted that the average body weight of the mice in the proguanil-treated group did not decrease during the treatment period (Fig. 8F, SFig. 4F). Similarly, no liver and kidney toxicity after proguanil treatment were observed as well (Fig. 8G, SFig. 4G). Thus, these results indicated that proguanil blocked BC tumor growth without obvious hepatotoxicity and nephrotoxicity.

## Discussion

As one of the most clinically useful targets, EGFR has been applied as a gold standard for treating various cancer patients [29–31]. However, rapidly acquired resistance and limited patient range have largely restricted its clinical application. Recently, targeting EGFR degradation is considered a new cancer treatment strategy [32–34]. Studies show that when internalized by NCE (non-Clathrin endocytosis), EGFR is degraded by ubiquitination, resulting in the reduction of EGFR expression on the membrane surface to block continuous signal transduction [35]. Feldman et al found that ursodeoxycholic acid enhanced EGFR endocytosis, ubiquitination and degradation to inhibit the growth of colon tumor cells by NCE [36]. In contrast, EGFR internalized by CME (Clathrin-mediated endocytosis) could enhance the EGFR signaling pathway since EGFR is recycling to the surface of the cell membrane. However, this recycling step could also be blocked by small molecules or antibodies, resulting in the decreased EGFR signaling transduction [17, 37–39]. Therefore, inducing EGFR degradation will beneficial for the treatment of EGFR high-expressing cancer patients. In this study, we found that proguanil can inhibit the proliferation of BC cells by directly binding to EGFR and enhancing its endocytosis and degradation, which provides a new thought for the clinical treatment of EGFR high expressing patients. However, how proguanil caused the decrease of EGFR expression and inhibited cell proliferation? First, we observed the effect of proguanil on the endocytic and degradation of EGFR after si Clathrin. The results showed that the endocytosis and degradation of EGFR enhanced by proguanil did not change after si Clathrin compared with si Ctrl group, while the ability that proguanil enhances EGFR endocytosis and degradation was significantly decreased after si Caveolin-1. Therefore, the possibility that proguanil promoted EGFR endocytosis through CME and then prevented EGFR recycling is excluded, suggesting the presence of another manner. In order to explore it, we demonstrated by CO-IP and found that the binding of EGFR to Caveolin-1 rather than Clathrin was enhanced after proguanil treatment. Further, another protein, c-Cbl plays a key role in substrate recruitment of ubiquitin. After silencing c-Cbl, proguanil-enhanced EGFR degradation was significantly reduced. These data revealed that c-Cbl is also critical in proguanil-enhanced EGFR degradation. We next explored whether proguanil affected EGFR-mediated downstream signaling pathways. WB results indicated that the phosphorylation of AKT/mTOR and c-Raf/ERK1/2 were significantly decreased after proguanil treatment. Previously, EGFR and its downstream signaling pathways were reported to regulate autophagy to influence tumor progression [28]. We found that autophagy was significantly increased after proguanil treatment. Furthermore, proguanil-induced autophagy was further enhanced in overexpressing EGFR cells. Interestingly, proguanil-induced autophagic death in BC cells was further enhanced after AKT/mTOR and ERK1/2 were blocked by inhibitors. these results suggest that proguanil-induced low expression of EGFR and inhibited phosphorylation of AKT/mTOR/c-Raf/ERK1/2 were the main driver of autophagy. Encouragingly, proguanil also has low toxicity at the antitumor dose because the weight loss of mice in the treatment group was not observed. In summary, this work has identified that proguanil specifically bound to EGFR and promoted its endocytosis through NCE, and then recruited c-Cbl to promote EGFR ubiquitination and degradation to induce autophagy to inhibit cell proliferation. Thus, this work may provide a mechanistic basis of proguanil for the future clinical treatment of EGFR high expressing BC patients.

## Materials and methods

### Cell culture and reagents

Bladder cancer cell lines, T24, J82, UMUC3, 5637, were obtained from iCell Bioscience Inc (Shanghai, China) and validated for authentication using the short tandem repeat (STR) method. All cells were performed in 5A (iCell, Shanghai, China), MEM (Gibco, Grand Island, NY) or RPMI-1640 (Hyclone Logan, UT, USA) added in 10% FBS (Hyclone, USA) and 1% penicillin/streptomycin at incubator (37 °C, 5% CO_2_). Antibodies and chemicals used in this study were listed in Supplementary Table 1.

### Human urothelial carcinoma tissues

Urothelial carcinoma tissues samples were used as previously described [20] Consents from all patients were obtained. Approval of this study was obtained from the Research Ethics Board at Xiangya Hospital (No. 201703229).

### SPRi affinity analysis

To explored the the target of proguanil, we performed an affinity measurement using surface plasmon resonance (SPRi) technology. In brief, the photocross-linking SensorChip was used to fix proguanil, T24 cell lysates circulated on the surface chip of chip, and the process of capturing target protein on the chip surface was detected by SPRi real time. The possible target proteins captured by proguanil were identified by LC-MS. Data collection and analysis was conducted by BetterWays (Guangzhou, China).

### MTT assay

Cells were plated in 96-well plates (6.0 × 10^3^/well), After 12 h, cells were treated or not treated with proguanil for 72 h. Subsequently, MTT Sigma-Aldrich (St. Louis, MO) solution (2 mg/mL, 50 μL) was transferred to wells and incubated for 5 h. Lastly, 150 μL dimethylsulfoxide (DMSO) was added in plates and the absorbance was gauged at 490 nm by using microplate reader (Biotek, SYNERGY HTX, VT, USA).

### Clonogenic assay

Cells were plated in 24-well plates (2.0 × 10^3^ /well), After 12 h, cells were treated or not treated with proguanil and let them to grow for 6–8 days. Subsequently, 10% formaldehyde solution was added to wells to fix the cells. Last, 0.1% crystal violet was transferred to visualize the colonies. Absorbance was gauged at 550 nm with microplate reader (Biotek, USA). The data were normalized to load Vehicle control.

### Western blot

Protein extracts were resolved through SDS-PAGE which then transferred to PVDF membranes, and probed with primary antibodies. Peroxidase-conjugated anti-mouse or rabbit antibody was used as secondary antibody and the antigen-antibody reaction was visualized by ChemiDoc system (Bio-Rad, Hercules, CA, USA). The commercial antibodies were used as show in Supplementary Table 1. The blot band intensities were quantitated by Image J. The data were normalized to load Vehicle control with the antibody against β-actin. The original images of all western blots were displayed in Original data file.

### Measurement of cell migration

4 × 10^4^ cells suspended in 200 μL non-FBS cell culture medium and were added to the upper chamber and 500 μL culture medium containing 10% FBS was added to the lower chamberof a Transwell plate (Corning, Shanghai, China) in the absence or presence of proguanil. After 24 h, 4% formaldehyde solution was used to fixed cells, Subsequently, 0.1% crystal violet was transferred to visualize and stained cells. The membranes were cleaned, air-dried, and photographed with a DFC450C microscope (Leica). The data were normalized to load Vehicle control.

### Immunoprecipitation

10^7^ cells are collected and lysed with lysis buffer (Beyotime, Shanghai, China). After 30 min of ice incubation, the lysate was centrifuged and the supernatant was collected. Then the supernatant was blocked by protein A/G (Santa Cruz, Dallas, TX) for 1 h. After centrifugation again, appropriate antibodies or Normal IgG were added to the supernatant and incubated overnight at 4 °C. Samples were incubated overnight. The protein A/G were added again to bind the antibody and collected by centrifugation. Last, the beads were washed four times using IP buffer. Sample loading buffer (2×) was mixed with the beads and boiled for 10 min. The supernatant was used for western blot analysis.

### RNA extraction and quantitative real-time-PCR (qRT-PCR)

RNA was isolated for qRT-PCR was performed using Trizol reagent. Complementary DNA (cDNA) was synthesized using a high-capacity cDNA reverse transcription kit (Thermo, Shanghai, China). qRT‐PCR was carried out using a TaqMan Gene Expression Master Mix (Bio‐Rad, Shanghai, China) according to manufacture protocol. The sequences of primers is shown in Supplementary Table 2.

### siRNA transfection

Cells were transfected with commercially available siRNA or negative control (NC) siRNA (Ribobio, =Shanghai, China) with the transfection reagent Lipofectamine 6000 (Invitrogen, Eugene, USA). Briefly, cells were plated in 6-well plates (2.0 × 10^5^/well), After 12 h, cells were transfected with 50 nM RNAi oligonucleotides and 50 nM Negative Control siRNA using Lipofectamine 6000 in the absence of FBS for 5 h. Following a washing in PBS, the medium was replaced with 5 A for an additional 24 h. Cell protein was collected and specific silencing was confirmed by WB and the sequences of siRNA are shown in Supplementary Table 3.

### Knockdown or overexpression of EGFR by transfection lentiviral vectors

The EGFR knockdown lentiviral vectors shCtrl, shEGFR-1, shEGFR-2, shEGFR-3 (Supplementary Table 4), and EGFR overexpression lentiviral vectors overexpression Ctrl (OC), overexpression EGFR (OE) were constructed by GeneChem Co. Ltd. (Shanghai, China). Briefly, cells were plated in 6-well plates (2.0 × 10^5^/well). After 12 h, the lentiviruses were added to the wells with 1 mL of 5A and 40 μl Transfection reagent (GeneChem, Shanghai, China) to incubate the cells for 12 h. Following a washing in PBS, the medium was replaced with 5A for an additional 72 h. Last, puromycin was added to the cell culture flask to kill untransfected cells.

### Monodansylcadaverine (MDC) staining

In order to investigate the autophagy in T24 and J82 cells, MDC kit (KeyGEN BioTECH, China) was utilized according to the manufacturer’s instruction. After cells in each group were treated with proguanil for corresponding time, it cleaned with 1× wash buffer. Subsequently, cells were incubated with MDC staining solution in darkness at room temperature for 45 min. After incubation, cells were washed three times with 1× wash buffer. Finally, cells were covered with100 μL of collection buffer and observed under a fluorescence microscope immediately.

### Subcutaneous xenograft model study

Four- to six-week-old female BALB/c nude mice (*n* = 35) were purchased from Hunan SJA Laboratory Animal Co., Ltd (Changsha, China). The study protocol was approved by the Ethics Committee of Hunan Normal University (D2020007). The mice were randomly assigned to groups. Mice were injected subcutaneously with shCtrl-T24 or ShEGFR-T24 cell suspension to construct xenograft models. Random animals were assigned to each treatment group. Tumor volumes were calculated according to the formula: 1/2 × long diameter × short diameter^2^. When the tumor grew to 70–100 cm^3^, the mice are treated with drugs or vehicle (2% PEG-400 + 2% Tween-80 + 96% PBS) through intraperitoneal injection. Tumor volumes and mouse weight were gauged every two days. After 14 days of proguanil treatment, the mice were killed. The liver and kidney were paraffin-embedded and sectioned and analyzed by HE. Finally, all tumors were kept in formalin for Ki-67 and immunohistochemistry. Investigators were blinded to the group allocation when assessing the results.

### Immunofluorescence

Cells were seeded on glass coverslips, washed thrice with PBS and fixed in 4% paraformaldehyde solution. After washing with PBS, 0.2% Triton X-100 was used to make cells permeable. Following wash with PBS again, cells were incubated for 30 min with 4% BSA. The primary antibody was used to incubate cells at 4 °C for overnight. After wash thrice with PBS, DyLight 549 (Proteintech, Chicago, USA) or Alexfluor 488 (Proteintech, USA) labeled secondary antibody was added to glass and incubated for 1 h. Nuclei were stained with DAPI and then fixed with glycerin and photographed under a fluorescence microscope. In Immunofluorescence staining analysis, data were calculated by Image J. The data were normalized to load Vehicle control.

### Histology

Tissues were fixed with 4% paraformaldehyde for 24 h, then embedded in paraffin and sectioned to 7 μm. Sections were stained with haematoxylin and eosin (H&E) and graded for disease. For immunohistochemistry staining, sections were deparaffinised and rehydrated using xylene, 100%, 95% then 75% ethanol. Subsequently, sections were incubated with 3% H_2_O_2_ for 20 min to block endogenous peroxidase activity, washed with PBS, and boiled in Tris-EDTA retrieval solution for 5 min in a pressure cooker. Then sections were incubated with primary antibody anti-Ki-67, anti-EGFR, anti-LC-3 overnight at 4 °C. Sections were washed with PBS again and incubated with Reagent 2 and Reagent 3 (Goat hypersensitivity two-step detection kit (ZSGB-BIO, Beijing, China)) according to manufacturers’instructions. Sections were stained by DAB Substrate Kit (Cell Signaling, Beverly, MA, USA) and counterstained with Gill’s haematoxylin (Solarbio, Beijing, China). Last, sections were then dehydrated and mounted using neutral resins (Solarbio, China). In IHC staining analysis, data were calculated by Image J. The data were normalized to load Vehicle control.

### Statistical analyses

The data of three independent experiments were expressed as mean ± SD. Statistical analysis was performed with SPSS 20.0. A statistical analysis was performed by Student’s *t*-test. We statistically compared the similar variances between the groups as well. All experiments were repeated at least three times. *P* < 0.05 was thought as statistically significant.

## Supplementary information

Supplementary information

Original data file

Data availability

Author contribution statement

Ethics declarations.

Conflict of interests

aj-checklist

## Data Availability

All data are available upon request.

## References

[1] Pfannstiel C, Strissel PL, Chiappinelli KB, Sikic D, Wach S, Wirtz RM (2019). The tumor immune microenvironment drives a prognostic relevance that correlates with bladder cancer subtypes. Cancer Immunol Res.

[2] Chauhan PS, Chen K, Babbra RK, Feng W, Pejovic N, Nallicheri A (2021). Urine tumor DNA detection of minimal residual disease in muscle-invasive bladder cancer treated with curative-intent radical cystectomy: a cohort study. PLoS Med.

[3] Bree KK, Hensley PJ, Westerman ME, Kokorovic A, Nogueras-Gonzalez GM, Dinney CP (2021). Contemporary rates of gynecologic organ involvement in females with muscle invasive bladder cancer: a retrospective review of women undergoing radical cystectomy following neoadjuvant chemotherapy. J Urol.

[4] Chang E, Weinstock C, Zhang L, Charlab R, Dorff SE, Gong Y (2021). FDA approval summary: enfortumab vedotin for locally advanced or metastatic urothelial carcinoma. Clin Cancer Res.

[5] Bahleda R, Italiano A, Hierro C, Mita A, Cervantes A, Chan N (2019). Multicenter Phase I study of erdafitinib (JNJ-42756493), oral pan-fibroblast growth factor receptor inhibitor, in patients with advanced or refractory solid tumors. Clin Cancer Res.

[6] Cathomas R, Lorch A, Bruins HM, Compérat EM, Cowan NC, Efstathiou JA (2021). The 2021 updated European Association of Urology Guidelines on Metastatic Urothelial Carcinoma. Eur Urol.

[7] Chow NH, Chan SH, Tzai TS, Ho CL, Liu HS (2001). Expression profiles of ErbB family receptors and prognosis in primary transitional cell carcinoma of the urinary bladder. Clin Cancer Res.

[8] Rebouissou S, Bernard-Pierrot I, de Reyniès A, Lepage ML, Krucker C, Chapeaublanc E (2014). EGFR as a potential therapeutic target for a subset of muscle-invasive bladder cancers presenting a basal-like phenotype. Sci Transl Med.

[9] Pruthi RS, Nielsen M, Heathcote S, Wallen EM, Rathmell WK, Godley P (2010). A phase II trial of neoadjuvant erlotinib in patients with muscle-invasive bladder cancer undergoing radical cystectomy: clinical and pathological results. BJU Int.

[10] Wong YN, Litwin S, Vaughn D, Cohen S, Plimack ER, Lee J (2012). Phase II trial of cetuximab with or without paclitaxel in patients with advanced urothelial tract carcinoma. J Clin Oncol.

[11] Lea MA, Kim H, des BC (2018). Effects of biguanides on growth and glycolysis of bladder and colon cancer cells. Anticancer Res.

[12] Linder M, Hecking M, Glitzner E, Zwerina K, Holcmann M, Bakiri L (2018). EGFR controls bone development by negatively regulating mTOR-signaling during osteoblast differentiation. Cell Death Differ.

[13] Voldborg BR, Damstrup L, Spang-Thomsen M, Poulsen HS (1997). Epidermal growth factor receptor (EGFR) and EGFR mutations, function and possible role in clinical trials. Ann Oncol.

[14] Pascolutti R, Algisi V, Conte A, Raimondi A, Pasham M, Upadhyayula S (2019). Molecularly distinct clathrin-coated pits differentially impact EGFR fate and signaling. Cell Rep.

[15] Renard HF, Boucrot E (2021). Unconventional endocytic mechanisms. Curr Opin Cell Biol.

[16] Sigismund S, Argenzio E, Tosoni D, Cavallaro E, Polo S, Di Fiore PP (2008). Clathrin-mediated internalization is essential for sustained EGFR signaling but dispensable for degradation. Dev Cell.

[17] Zhao L, Qiu T, Jiang D, Xu H, Zou L, Yang Q (2020). SGCE promotes breast cancer stem cells by stabilizing EGFR. Adv Sci.

[18] Lonic A, Gehling F, Belle L, Li X, Schieber NL, Nguyen EV (2021). Phosphorylation of PKCδ by FER tips the balance from EGFR degradation to recycling. J Cell Biol.

[19] Zhang H, Han B, Lu H, Zhao Y, Chen X, Meng Q (2018). USP22 promotes resistance to EGFR-TKIs by preventing ubiquitination-mediated EGFR degradation in EGFR-mutant lung adenocarcinoma. Cancer Lett.

[20] Deng J, Peng M, Zhou S, Xiao D, Hu X, Xu S (2021). Metformin targets Clusterin to control lipogenesis and inhibit the growth of bladder cancer cells through SREBP-1c/FASN axis. Signal Transduct Target Ther.

[21] Xiao D, Lu Z, Wang Z, Zhou S, Cao M, Deng J (2020). Synthesis, biological evaluation and anti-proliferative mechanism of fluorine-containing proguanil derivatives. Bioorg Med Chem.

[22] Zhong L, Liao D, Zhang M, Zeng C, Li X, Zhang R (2019). YTHDF2 suppresses cell proliferation and growth via destabilizing the EGFR mRNA in hepatocellular carcinoma. Cancer Lett.

[23] Mooso BA, Vinall RL, Mudryj M, Yap SA, deVere White RW, Ghosh PM (2015). The role of EGFR family inhibitors in muscle invasive bladder cancer: a review of clinical data and molecular evidence. J Urol.

[24] Huang KY, Kao SH, Wang WL, Chen CY, Hsiao TH, Salunke SB (2016). Small molecule T315 promotes casitas B-lineage lymphoma-dependent degradation of epidermal growth factor receptor via Y1045 autophosphorylation. Am J Respir Crit Care Med.

[25] Belizaire R, Koochaki SHJ, Udeshi ND, Vedder A, Sun L, Svinkina T (2021). CBL mutations drive PI3K/AKT signaling via increased interaction with LYN and PIK3R1. Blood..

[26] Sooro MA, Zhang N, Zhang P (2018). Targeting EGFR-mediated autophagy as a potential strategy for cancer therapy. Int J Cancer.

[27] Wu M, Zhang P (2020). EGFR-mediated autophagy in tumourigenesis and therapeutic resistance. Cancer Lett.

[28] Si Y, Zhang H, Peng P, Zhu C, Shen J, Xiong Y (2021). G protein pathway suppressor 2 suppresses gastric cancer by destabilizing epidermal growth factor receptor. Cancer Sci.

[29] Ma J, Ma S, Zhang Y, Shen Y, Huang L, Lu T (2021). Kinectin1 depletion promotes EGFR degradation via the ubiquitin-proteosome system in cutaneous squamous cell carcinoma. Cell Death Dis.

[30] Li T, Tao Z, Zhu Y, Liu X, Wang L, Du Y (2021). Exosomal annexin A6 induces gemcitabine resistance by inhibiting ubiquitination and degradation of EGFR in triple-negative breast cancer. Cell Death Dis.

[31] Kumagai S, Koyama S, Nishikawa H (2021). Antitumour immunity regulated by aberrant ERBB family signalling. Nat Rev Cancer.

[32] Dokla EME, Fang CS, Abouzid KAM, Chen CS (2019). 1,2,4-Oxadiazole derivatives targeting EGFR and c-Met degradation in TKI resistant NSCLC. Eur J Med Chem.

[33] Ménard L, Floc’h N, Martin MJ, Cross DAE (2018). Reactivation of mutant-EGFR degradation through clathrin inhibition overcomes resistance to EGFR tyrosine kinase inhibitors. Cancer Res.

[34] Cheng M, Yu X, Lu K, Xie L, Wang L, Meng F (2020). Discovery of potent and selective epidermal growth factor receptor (EGFR) bifunctional small-molecule degraders. J Med Chem.

[35] Tanaka T, Zhou Y, Ozawa T, Okizono R, Banba A, Yamamura T (2018). Ligand-activated epidermal growth factor receptor (EGFR) signaling governs endocytic trafficking of unliganded receptor monomers by non-canonical phosphorylation. J Biol Chem.

[36] Feldman R, Martinez JD (2009). Growth suppression by ursodeoxycholic acid involves caveolin-1 enhanced degradation of EGFR. Biochim Biophys Acta.

[37] Friedman LM, Rinon A, Schechter B, Lyass L, Lavi S, Bacus SS (2005). Synergistic down-regulation of receptor tyrosine kinases by combinations of mAbs: implications for cancer immunotherapy. Proc Natl Acad Sci USA.

[38] Perera RM, Narita Y, Furnari FB, Gan HK, Murone C, Ahlkvist M (2005). Treatment of human tumor xenografts with monoclonal antibody 806 in combination with a prototypical epidermal growth factor receptor-specific antibody generates enhanced antitumor activity. Clin Cancer Res.

[39] Pedersen MW, Jacobsen HJ, Koefoed K, Hey A, Pyke C, Haurum JS (2010). Sym004: a novel synergistic anti-epidermal growth factor receptor antibody mixture with superior anticancer efficacy. Cancer Res.

