# Supplementary material for: Inhibitory role of proguanil on the growth of bladder cancer via enhancing EGFR degradation and inhibiting its downstream signaling pathway to induce autophagy
Source: Cell Death Dis. 2022 May 25;13(5):499. doi: 10.1038/s41419-022-04937-z (PMC9132982; doi:10.1038/s41419-022-04937-z)
Supplement: Supplementary file 1 — Supplementary information [file 41419_2022_4937_MOESM1_ESM.docx]

**Supplementary information**

**Inhibitory role of proguanil on the growth of bladder cancer via enhancing EGFR degradation and inhibiting its downstream signaling pathway to induce autophagy**

Di Xiao^1, 3^, Xin Hu^1, 3^, Mei Peng^1^, Jun Deng^1^, Sichun Zhou^1^, Simeng Xu^1^, Jingtao Wu^1^, Xiaoping Yang^1, 2*^

^1^Key Laboratory of Study and Discovery of Small Targeted Molecules of Hunan Province, Department of Pharmacy, School of Medicine, Hunan Normal University, Changsha, Hunan, China. ^2^Key Laboratory of Protein Chemistry and Developmental Biology of Fish of Ministry of Education, Hunan Normal University, Changsha, Hunan, China. ^3^These authors contributed equally: Di Xiao, Xin Hu.

**Address for correspondence:**

Xiaoping Yang, Key Laboratory of Study and Discovery of Small Targeted Molecules of Hunan Province, Department of Pharmacy, School of Medicine, Hunan Normal University, Changsha, 410013 Hunan, China. Tel/Fax: 01186-158-7406-6132; Email: xiaoping.yang@hunnu.edu.cn

*Corresponding author

Tel/Fax: 01186-158-7406-6132

E-mail address：xiaoping.yang@hunnu.edu.cn (X. Yang)

**Contents**

Supplementary Tables………………………………………………………………..3

Supplementary Material and Methods……………………………………………….5

Supplementary Figure legends……………………………………………………….6

**Supplementary Table 1. Chemicals and primary antibodies used in this study**

| **Name** | **Supplier** | **Cat no.** |
| --- | --- | --- |
| Proguanil | Selleck | S5927 |
| 3-MA | MedChemExpress | HY-19312 |
| MK-2206 | MedChemExpress | HY-10358 |
| AY-22989 | MedChemExpress | HY-10219 |
| AZD6244 | MedChemExpress | HY-50706 |
| EGF | Cell Signaling Technology | 8916SC |
| biotinylated-EGF | Invitrogen | E3477 |
| streptavidin-POD | Roche | 11089153001 |
| O-phenylenediamine | Aladdin | P107882 |
| EGFR | Cell Signaling Technology | 4267 |
| p-EGFR | Cell Signaling Technology | 3777 |
| Caveolin-1 | Cell Signaling Technology | 3267 |
| Clathrin | Cell Signaling Technology | 4796 |
| p-mTOR | Cell Signaling Technology | 5536 |
| p-c-Raf | Cell Signaling Technology | 9421 |
| p-AKT | Cell Signaling Technology | 4060 |
| p-ERK | Cell Signaling Technology | 4370 |
| LC3 | Cell Signaling Technology | 12741 |
| Beclin1 | Cell Signaling Technology | 3738 |
| c-Cbl | santa | sc-1651 |
| Ubiquitin | R&D Systems | MAB701 |

**Supplementary Table 2. Primers used in this study**

| Gene | Forward Primer | Reverse Primer |
| --- | --- | --- |
| EGFR | GAATTCGATGATCAACTCACGG | ACCCATATGTACCATCGATGTC |

**Supplementary Table 3. The targeting oligos of Caveolin-1, Clathrin and c-Cbl.**

| siRNA | 5'-3' |
| --- | --- |
| Caveolin-1 | GGCCAGCUUCACCACCUUC |
| Clathrin | GAAGAACTCTTTGCCCGGAAATTTA |
| c-Cbl | CCUCUCUUCCAAGCACUGA |

**Supplementary Table 4. The targeting oligos of EGFR.**

| Sh EGFR-1 | CGCAAAGTGTGTAACGGAATA |
| --- | --- |
| Sh EGFR-2 | CATCAGTGGCGATCTCCACAT |
| Sh EGFR-3 | CTGGATCCACAGGAACTGGAT |

**Supplementary Material and Methods**

**Endocytosis Assay**

Cells were plated in 96-well plates (2.0×10^4^ /well) and incubated with 20 ng/ml of biotinylated-EGF (Invitrogen, Eugene, USA) in assay buffer (PBS4+: PBS supplemented with 1 mM MgCl2, 1 mM CaCl2, 5 mM glucose and 0.2% bovine serum albumin) at 37℃ for the indicated time points. Cells were then immediately cooled down (4℃) to stop internalization. The biotinylated-EGF was removed from the cell surface by an acid wash step (0.2 M acetic acid, 0.2 M NaCl, pH 2.5). Cells were then washed with cold PBS and fixed in 4% paraformaldehyde for 30 min and further permeabilized with 0.1% Triton X-100 for 10 min. Internalized biotinylated-EGF was assessed by streptavidin-POD (Roche). The reaction was further developed with O-phenylenediamine (Aladdin, Shanghai, China), and then stopped by addition of 50 μl of 5M of H_2_SO_4_. The absorbance was read at 490 nm with microplate reader (Biotek, USA).

**Supplementary Figure Legends**

**SFig. 1 Proguanil mediates EGFR degradation in the lysosome.** A: T24 were plated in 6-well plates (3.0×10^5^ /well). When the cell density reaches 70%-80%, cells were starved for 12h and then treated with proguanil for 6 hours. The changes of EGFR and its downstream signal pathways were detected by WB experiment. B: T24 were plated in 6-well plates (3.0×10^5^ /well). When the cell density reaches 70%-80%, cells were starved for 12h and then pretreated with chloroquine for 12 hours to destroy the lysosomes, and then treated with proguanil for 6 hours. The changes of EGFR and its downstream signal pathways were detected by WB experiment. Data are representative of three independent experiments. Error bars represent means ± SD from triplicate experiments. Vehicle control means the concentration of DMSO lower than 0.3% (*P<0.05, **P< 0.01, ***P<0.001).

**SFig. 2 Proguanil blocked EGF-induced EGFR endocytosis and inhibits EGFR downstream signaling pathways.** A: The endocytosis of EGFR induced by EGF were detected by Endocytosis assay as described in Materials and Methods. B: T24 were plated in 6-well plates (3.0×10^5^ /well). When the cell density reaches 70%-80%, cells is stimulated by EGF for 30 minutes, and the changes of EGFR and its downstream signal pathways were detected by WB experiment. C: T24 were plated in 6-well plates (3.0×10^5^ /well). When the cell density reaches 70%-80%, cells were starved for 12 hours, and then treated with proguanil for 6 hours. The endocytosis of EGFR was detected by Endocytosis assay as described in Materials and Methods. D: T24 were plated in 6-well plates (3.0×10^5^ /well). When the cell density reaches 70%-80%, cells were starved for 12 hours, treated with proguanil for 6 hours and then stimulated by EGF. The changes of EGFR and its downstream signal pathways were detected by WB experiment. Data are representative of three independent experiments. Error bars represent means ± SD from triplicate experiments. Vehicle control means the concentration of DMSO lower than 0.3% (*P<0.05, **P< 0.01, ***P<0.001).

**SFig. 3 Proguanil blocked ligand-induced activation of EGFR signaling pathway.** A, C: T24 were plated in 96-well plates (6.0×10^3^ /well) and pretreated with 400nm MK-2206 or AY-22989 for 12 h before exposure to increasing concentrations of proguanil for an additional 72 h, the combined effect of proguanil and MK-2206 or AY-22989 was detected by MTT assay. B, D: Combination index (CI) among the combinations of two drugs was calculated using CompuSyn software. if CI >1, it denotes antagonism; if CI <1, it denotes synergism. CI values in all of combinations were less than 1, indicating synergism. E-F: T24 were plated in 6-well plates (3.0×10^5^ /well). When the cell density reaches 70%-80%, cells were starved for 12h and then pretreated with 400nm MK-2206 or AY-22989 for 12 h before exposure to proguanil for an additional 12 h, the protein expressions of p-AKT, p-mTOR and LC3 were detected by WB. Data are representative of three independent experiments. Error bars represent means ± SD from triplicate experiments. Vehicle control means the concentration of DMSO lower than 0.3% (*P<0.05, **P< 0.01, ***P<0.001).

**SFig. 4 Proguanil enhanced EGFR degradation to induce autophagy to inhibit the growth of xenograft tumors in vivo.** A: 10^7^ T24 were injected into the right axilla of mice. When the tumor volume reached 70-100 mm^3^, mice were treated with proguanil and 3MA. After treated 14 days, tumors were removed and photographed. B: The tumor volume of each mouse was measured every two days, and the mean volume of each group tumor was calculated to create the figure. C: Statistical analysis of tumor weight. D: Ki-67 was used to analyze the proliferation of xenograft tumor. E: The expression of LC3 in tumor tissues was detected by immunohistochemistry. F: Changes of each group mice weight. G: HE staining of liver and kidney organs of each group mice. All in vitro experiments are representative of three independent experiments. Error bars represent means ± SD. Vehicle control(2％PEG-400+2％Tween-80+96％PBS) (*P<0.05, **P<0.01, ***P<0.001).
