# Supplementary figures and images for: Inhibitory role of proguanil on the growth of bladder cancer via enhancing EGFR degradation and inhibiting its downstream signaling pathway to induce autophagy
Source: Cell Death Dis. 2022 May 25;13(5):499. doi: 10.1038/s41419-022-04937-z (PMC9132982; doi:10.1038/s41419-022-04937-z)

**Supplementary figure 1**


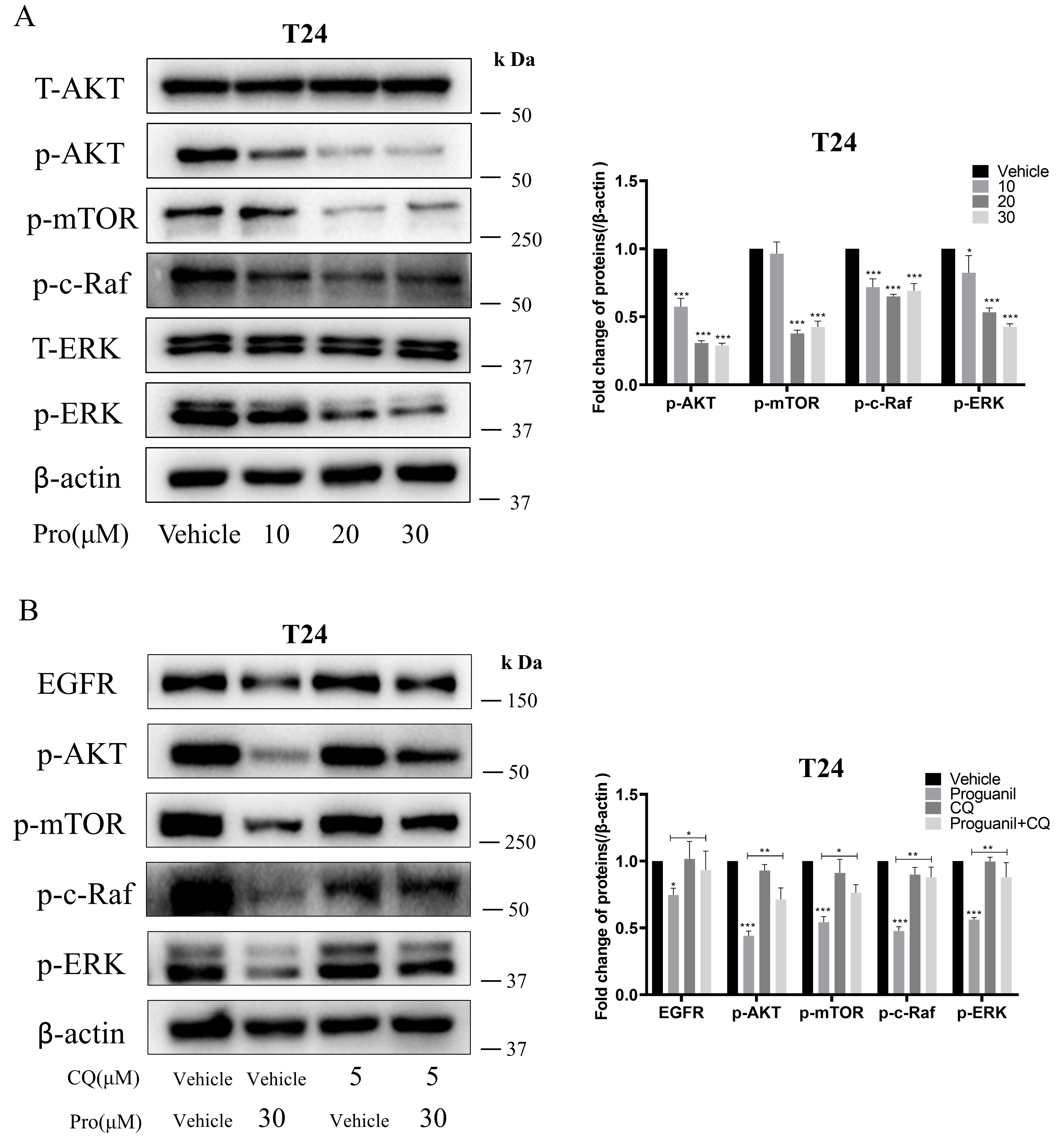


**Supplementary figure 2**


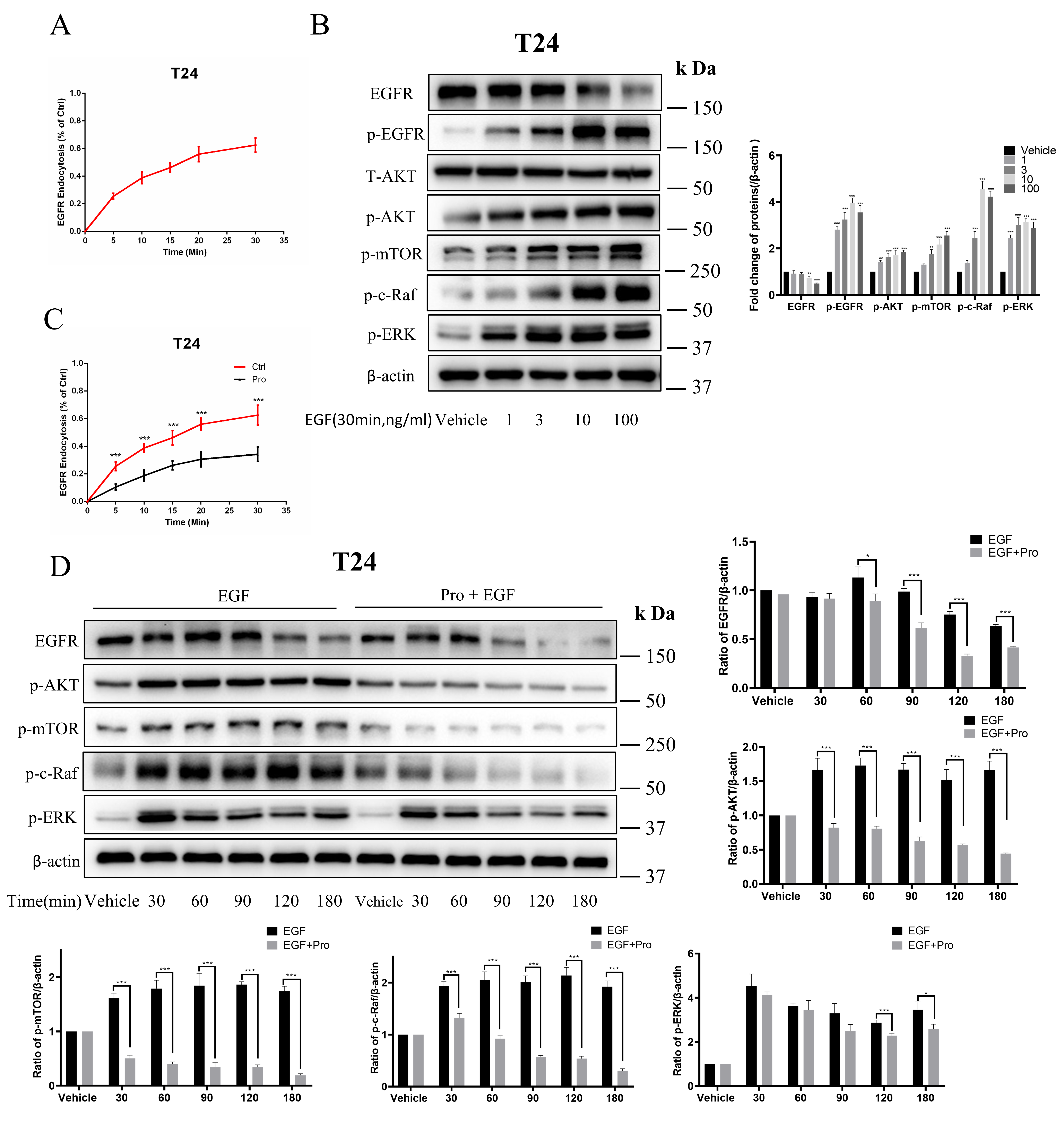


**Supplementary figure 3**


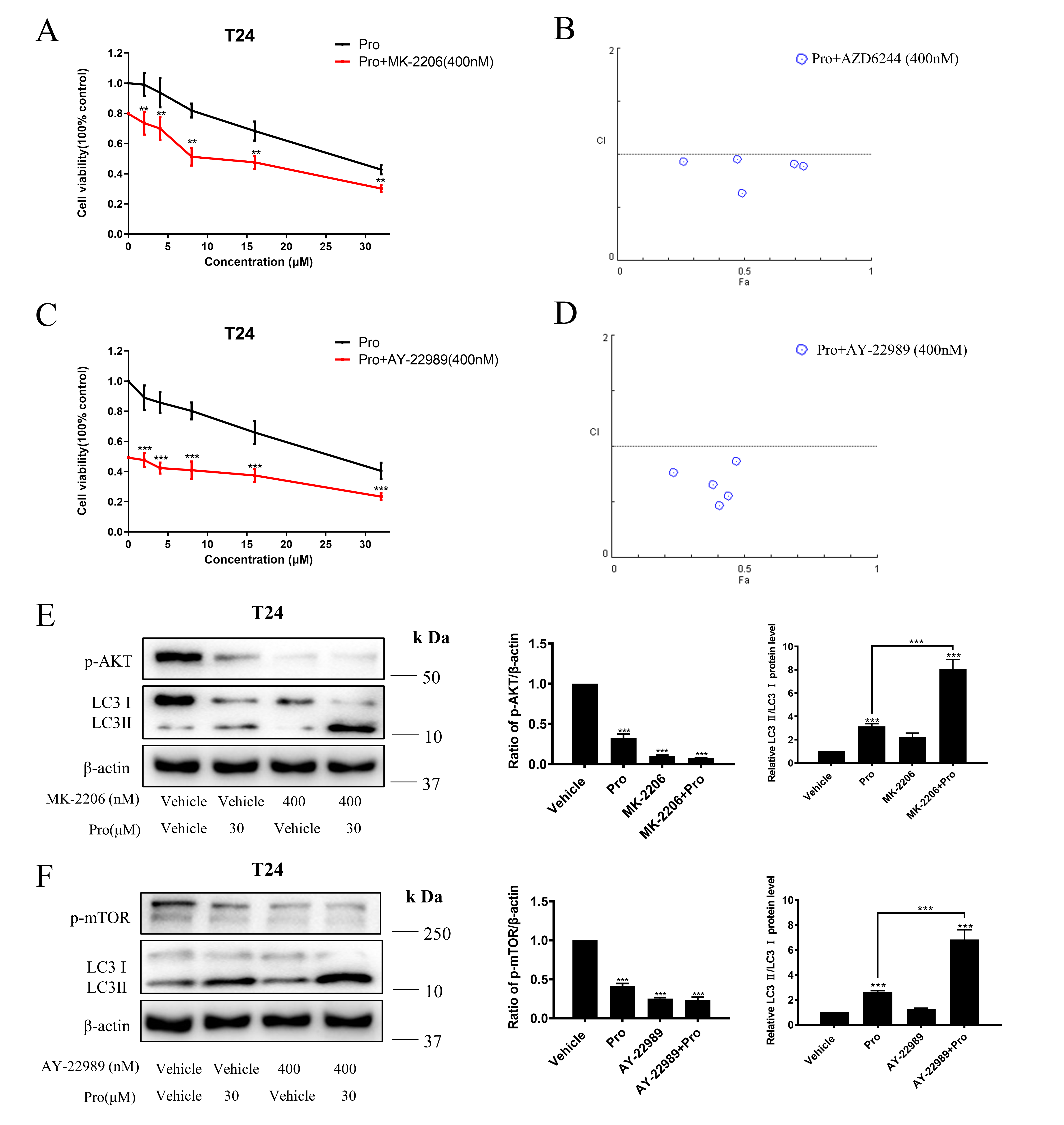


**Supplementary figure 4**


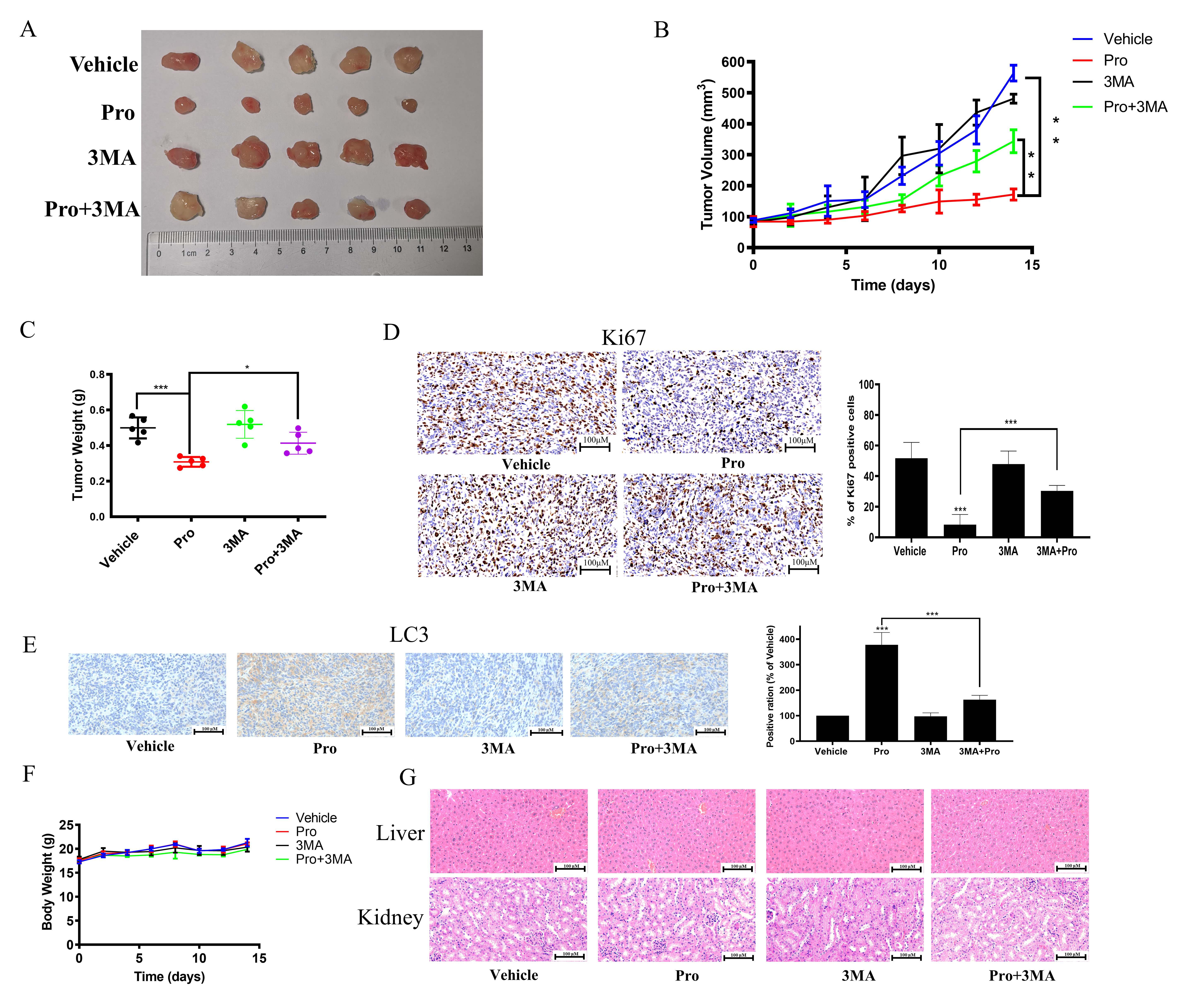

Supplement: Supplementary file 2 — Original data file [file 41419_2022_4937_MOESM2_ESM.docx]
