# Supplementary material for: Inhibitory role of proguanil on the growth of bladder cancer via enhancing EGFR degradation and inhibiting its downstream signaling pathway to induce autophagy
Source: Cell Death Dis. 2022 May 25;13(5):499. doi: 10.1038/s41419-022-04937-z (PMC9132982; doi:10.1038/s41419-022-04937-z)
Supplement: Supplementary file 3 — Data availability [file 41419_2022_4937_MOESM3_ESM.docx]

**Data availability**

All data are available upon request.
