# Supplementary material for: Inhibitory role of proguanil on the growth of bladder cancer via enhancing EGFR degradation and inhibiting its downstream signaling pathway to induce autophagy
Source: Cell Death Dis. 2022 May 25;13(5):499. doi: 10.1038/s41419-022-04937-z (PMC9132982; doi:10.1038/s41419-022-04937-z)
Supplement: Supplementary file 4 — Author contribution statement [file 41419_2022_4937_MOESM4_ESM.docx]

**AUTHOR CONTRIBUTIONS**

Di Xiao and Xin Hu conducted the experiments, interpreted results and drafted the manuscript. Mei Peng prepared and calculated the data. Jun Deng, Sichun Zhou, Simeng Xu, Jingtao Wu participated in sample and data collection. Xiaoping Yang designed the study, edited the manuscript, and participated in interpretation of the results. All authors read and approved the final manuscript.
