# Supplementary material for: Inhibitory role of proguanil on the growth of bladder cancer via enhancing EGFR degradation and inhibiting its downstream signaling pathway to induce autophagy
Source: Cell Death Dis. 2022 May 25;13(5):499. doi: 10.1038/s41419-022-04937-z (PMC9132982; doi:10.1038/s41419-022-04937-z)
Supplement: Supplementary file 5 — Ethics declarations. [file 41419_2022_4937_MOESM5_ESM.docx]

**Ethics statement**

**Competing interests**

The authors declare no competing interests.

**Ethics**

Urothelial carcinoma tissues in this study were approved by Ethical Committee of Xiangya Hospital (No. 201703229). Animal experiment was approved by the Ethics Committee of Hunan Normal University (D2020007)..
